# Myotome adaptability confers developmental robustness to somitic myogenesis in response to fibre number alteration

**DOI:** 10.1016/j.ydbio.2017.08.029

**Published:** 2017-11-15

**Authors:** Shukolpa D. Roy, Victoria C. Williams, Tapan G. Pipalia, Kuoyu Li, Christina L. Hammond, Stefanie Knappe, Robert D. Knight, Simon M. Hughes

**Affiliations:** a Randall Division of Cell and Molecular Biophysics, New Hunt's House, Guy's Campus, King's College London, London SE1 1UL, UK; b Division of Craniofacial Development and Stem Cell Biology, Guy's Hospital, King's College London, UK

**Keywords:** Muscle, Zebrafish, Myosin, Myod, Myogenin, Pax7

## Abstract

Balancing the number of stem cells and their progeny is crucial for tissue development and repair. Here we examine how cell numbers and overall muscle size are tightly regulated during zebrafish somitic muscle development. Muscle stem/precursor cell (MPCs) expressing Pax7 are initially located in the dermomyotome (DM) external cell layer, adopt a highly stereotypical distribution and thereafter a proportion of MPCs migrate into the myotome. Regional variations in the proliferation and terminal differentiation of MPCs contribute to growth of the myotome. To probe the robustness of muscle size control and spatiotemporal regulation of MPCs, we compared the behaviour of wild type (wt) MPCs with those in mutant zebrafish that lack the muscle regulatory factor Myod. *Myod*^*fh261*^ mutants form one third fewer multinucleate fast muscle fibres than wt and show a significant expansion of the Pax7^+^ MPC population in the DM. Subsequently, *myod*^*fh261*^ mutant fibres generate more cytoplasm per nucleus, leading to recovery of muscle bulk. In addition, relative to wt siblings, there is an increased number of MPCs in *myod*^*fh261*^ mutants and these migrate prematurely into the myotome, differentiate and contribute to the hypertrophy of existing fibres. Thus, homeostatic reduction of the excess MPCs returns their number to normal levels, but fibre numbers remain low. The GSK3 antagonist BIO prevents MPC migration into the deep myotome, suggesting that canonical Wnt pathway activation maintains the DM in zebrafish, as in amniotes. BIO does not, however, block recovery of the *myod*^*fh261*^ mutant myotome, indicating that homeostasis acts on fibre intrinsic growth to maintain muscle bulk. The findings suggest the existence of a critical window for early fast fibre formation followed by a period in which homeostatic mechanisms regulate myotome growth by controlling fibre size. The feedback controls we reveal in muscle help explain the extremely precise grading of myotome size along the body axis irrespective of fish size, nutrition and genetic variation and may form a paradigm for wider matching of organ size.

## Highlights

• A critical window for early muscle fibre formation is proposed.
• Fish lacking MyoD1 form fewer muscle fibres, but have more myogenic stem cells.
• Stem cell numbers rapidly return to normal during subsequent development.
• GSK3 activity promotes and MyoD1 delays myoblast migration into the myotome.
• Compensatory fibre size increase ensures robustness of overall muscle size.

## Graphical abstract

**Figure:**
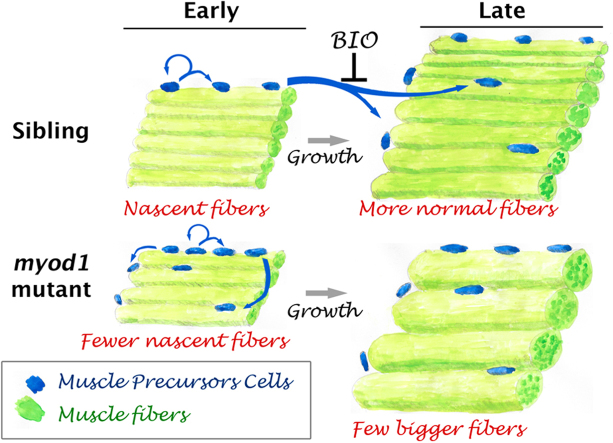

## 1 Introduction

How tissue size is regulated is largely unknown, but depends on both the number of cells and their size. When the ‘correct’ size is reached, growth ceases. Although signalling pathways such as IGF, BMP, TOR and Hippo have been implicated in tissue size control (Gokhale and Shingleton, 2015, Irvine and Harvey, 2015), general understanding is lacking. Closely related vertebrate species with distinct ploidy have long been known to alter cell size, yet maintain tissue size through a reduction in cell number (Cavalier-Smith, 2005, Fankhauser, 1945, Gokhale and Shingleton, 2015, Otto, 2007). Thus, tissues appear to measure their absolute size and regulate cell proliferation accordingly, rather than simply generating the correct cell number. Such findings suggest there is feedback regulation between tissue size and stem/precursor cell populations.

Skeletal muscle is a post-mitotic tissue that has the unusual capacity to change size during normal life. All body muscle derives from lineage-restricted stem/precursor cells called myoblasts, that originate from the somitic dermomyotome (Bentzinger et al., 2012). Growth involves three processes: formation of new fibres, fusion of additional myoblasts to existing fibres and increase in cell volume per nucleus. Surprisingly, the contribution of each to tissue growth has not been distinguished in previous studies of embryonic myogenesis. In mammals, fibre formation ceases shortly after birth (Ontell et al., 1988, Ontell and Kozeka, 1984). Fibre number can be a major determinant of muscle size; strains of sheep with different muscle sizes show corresponding differences in fibre number, but not fibre size (Bunger et al., 2009). How myoblasts choose whether to initiate a new fibre or fuse to an existing fibre is unclear. In *Drosophila*, distinct molecular pathways create founder myoblasts, which initiate fibres, and fusion competent myoblasts, which augment fibre growth (Abmayr and Pavlath, 2012). Our recent analyses of zebrafish muscle repair (Knappe et al., 2015, Pipalia et al., 2016) revealed two Pax7-expressing myoblast sub-populations with similarities to founder and fusion-competent cells (Pipalia et al., 2016). Whether such myoblast diversity underlies fibre formation during development and thereby determines muscle size in vertebrates is unknown.

Many genes have been implicated in differentiation and fusion of MPCs marked by Pax7 (Bentzinger et al., 2012). Among them are MyoD and Myogenin, members of the MyoD family of myogenic regulatory transcription factors (MRFs) that drive murine myoblast formation and muscle differentiation (Hasty et al., 1993, Nabeshima et al., 1993, Rawls et al., 1995, Rudnicki et al., 1993, Venuti et al., 1995). MyoD is required for the formation of specific populations of muscle cells early in development, but *Myod* mutants are viable (Kablar et al., 1997, Rudnicki et al., 1992, Tajbakhsh et al., 1997). In contrast, Myogenin appears to be required for differentiation of cells that normally contribute to fusion (Hasty et al., 1993, Nabeshima et al., 1993, Rawls et al., 1995, Venuti et al., 1995). After fibre formation, MRF levels within muscle fibres correlate negatively with fibre size and manipulations influence adult fibre size, particularly the response to neurogenic atrophy (Hughes et al., 1999, Moresi et al., 2010, Moretti et al., 2016). Thus, due to their pleiotropic roles, MRFs influence murine muscle size in complex ways.

As in amniotes, the zebrafish myotome forms by the terminal differentiation of myoblasts under the control of MRF genes (Hammond et al., 2007, Hinits et al., 2009, Hinits et al., 2011, Maves et al., 2007, Schnapp et al., 2009). In parallel with this process, cells in the anterior somite border generate a Pax3/7-expressing DM external cell layer (Devoto et al., 2006, Groves et al., 2005, Hammond et al., 2007, Hollway et al., 2007, Stellabotte and Devoto, 2007, Stellabotte et al., 2007). Cells of the DM appear to contribute to later muscle growth (Stellabotte et al., 2007). Lineage tracing of zebrafish DM cells suggests that they also contribute to fin, sternohyal and oesophageal muscles (Minchin et al., 2013, Neyt et al., 2000). However, quantitative mechanistic understanding of how DM cell dynamics are controlled within the somite and relate to later fibre formation is lacking.

We have previously shown that the zebrafish myotome rapidly increases in volume during the pre- and post-hatching period, growing threefold between 1 and 5 days post-fertilization (dpf) (Hinits et al., 2011). Zebrafish muscle shows size homeostasis in response to altered Myod activity. *Myod* mutants lack specific populations of early myogenic cells so that the myotome is reduced in size by 50% at 1 dpf (Hinits et al., 2009, Hinits et al., 2011). Nevertheless, the myotome of *myod* mutants grows rapidly, approaching normal size by 5 dpf (Hinits et al., 2011). We set out to discover how this happens.

After initial fibre formation in normal growth, dermomyotome-derived Pax7-expressing myogenic cells ingress into the deep myotome around 3 dpf, where a portion express Myogenin and differentiate into fibres, leading to a small increase in fibre number. *Myod* mutants have fewer fibres and fibre number fails to increase. Nevertheless, the remaining fibres grow larger than those in wt. Ingression of Pax7^+^ cells into the myotome is accelerated in *myod* mutants and more cells appear to differentiate. Inhibition of GSK3 activity prevents Pax7^+^ cell ingression, but does not diminish muscle size recovery in *myod* mutants, or block growth. The myotome thus responds to reduction in fibre number by hypertrophy of remaining fibres. Our data show that feedback between muscle fibres and their precursor cells regulates myotome growth and that although homeostasis in young animals recovers muscle mass it leaves a persistent alteration in fibre number.

## 2 Materials and methods

### 2.1 Zebrafish lines and maintenance

Genetically-altered *Danio rerio* (listed in Table 1) on a primarily AB background were reared at King's College London on a 14/10 h light/dark cycle at 28.5 °C (Westerfield, 2000). Adults were kept at 26.5 °C and embryos/larvae reared at 28.5 °C in the dark, except for periods outside the incubator. Throughout the current work, ages quoted as ‘N dpf‘ correspond to a developmental period at 28.5 °C of 24N + 0–8 hours, due to the need to scan live fish sequentially. BIO (0.5 μM; Tocris #3194) or DMSO vehicle were added to fish water. All experiments were performed in accordance with licences held under the UK Animals (Scientific Procedures) Act 1986 and later modifications and conforming to all relevant guidelines and regulations.

**Table 1:** Fish alleles.

**Fish Line**	**References**	**Notes**
*myod*^*fh261*^	(Hinits et al., 2011)	Likely null
*Tg(Ola.Actb:Hsa.HRAS-EGFP)*^*vu119*^	(Cooper et al., 2005)	Broadly/ubiquitously expressed and anchors EGFP to plasma membrane
*Tg(pax7a:EGFP)*^t32239Tg^	(Mahalwar et al., 2014)	A generous gift from C. Nüsslein-Volhard, MPI Tübingen, Germany. Recombineered BAC transgenic.
*pfeffer*^*tm236b*^	(Odenthal et al., 1996)	Mutation in *csf1ra*, which reduces the number of Pax7-expressing xanthophores
*Tg(pax7a:EGFP)*^t32239Tg^*;pfe*^*tm236/tm236*^	(Alsheimer, 2012)	Bred onto *pfeffer*^*tm236b*^ to aid observation of MPCs
*Tg(−2.2mylz2:GFP)*^*i135*^	(von Hofsten et al., 2008)	Marks fast muscle fibres

### 2.2 Immunodetection and S-phase labelling

Fibre sizes on photomicrographs of cryosections either unstained or after immunoperoxidase detection of MyHC (Table 2) were quantified with OpenLab (Improvision). For wholemounts, larval pigmentation was suppressed with 0.003% 1-phenyl-2-thiourea (Sigma) added at 12 hpf. Larvae were fixed with 2% PFA for 25 min, washed with PBTx (PBS, 0.5% or 1% (4 dpf+) Triton-X100) and incubated in primary antibody (see Table 2) for 3–5 days at 4 °C on a rotary shaker, washed repeatedly in PBTx, incubated with subclass specific Alexa-conjugated secondary antibodies (Molecular Probes) overnight, repeatedly washed with PBTx prior to incubation with 1 μM Hoechst 33342 for 2 h at room temperature. Larvae were washed and mounted under a cover slip with Citifluor AF1 for imaging. Larvae were S-phase labelled by exposure to 1 mg/ml EdU in 10% PBS:90% system water for 3 h, immediately fixed with 2% PFA and EdU detected with a Click-iT kit (Invitrogen C10084).

**Table 2:** Antibodies used.

Antigen	Dilution	Source	References	Antibody Registry
Pax7	1:5	DSHB	(Kawakami et al., 1997)	AB_528428
Myogenin	1:50	Santa Cruz Biotechnology sc-576		AB_2148908
eGFP	1:500	Abcam ab13970		AB_300798
General MyHCs	1:5	A4.1025	(Dan-Goor et al., 1990)	AB_528356
Slow MyHC	1:5	F59	(Devoto et al., 1996)	AB_528373
Fast fibre MyHC (Fig. 1A-C)	1:5	BA-F8	(Schiaffino et al., 1998)	AB_10572253
Slow fibre MyHC (Fig. 1A-C)	1:5	BA-D5	(Schiaffino et al., 1998)	AB_2235587
Non-intermediate fibre MyHC(s) (Fig. 1A-C)	1:5	SC71	(Schiaffino et al., 1998)	AB_2147165

### 2.3 Imaging and quantification

All images of fish are oriented dorsal up in either transverse or lateral anterior to left view. Larvae were anaesthetized with MS222, mounted in 1% low melting agarose and viewed laterally by a Zeiss 20×/1.0 NA dipping objective on an LSM Exciter confocal microscope with ZEN (2009 + 2012) software or a Nikon D-Eclipse C1 microscope with 40×/0.8 NA water dipping objective and EZ-C1 3.70 software. In Fig. 3A, Z-stacks were acquired in 1 µm steps from peri/epidermis to neural tube, processed with Fiji, drift adjusted with 'Correct 3D drift' and single cells tracked manually with 'MtrackJ'. To account for drift and growth, a reference point on the peri/epidermis was also tracked and the respective *Z*-value subtracted from that of individual *pax7a:GFP+* cells at each of the 28 time-points to obtain a depth measurement relative to peri/epidermis. Absolute movement in *Z* for each cell in Fig. 3B was calculated by subtracting the position at 82 hpf from that at each subsequent time-point.

Somite volume and fibre number were measured from confocal stacks of somite 17 in *Tg(Ola.Actb:Hsa.HRAS-EGFP)*^*vu119*^ larvae as described (Hinits et al., 2011). Mean fibre number (fast plus slow) was calculated from three optical sections after correcting for double counting at vertical myosepta (VM) using Fibre number = Total fibre profiles – (Profiles touching VM)/2. Mean fibre volume = Myotome volume/Mean fibre number.

Fixed fish were imaged using the 10×/0.3 air or 40×/1.1 water immersion objectives. Three to nine somites around the anal vent were imaged from lateral using the tile scan *Z*-stack function. Short stack maximum intensity projections, specific slices or cross-sectional views were exported as tiffs. Nuclear number was determined from three equi-spaced transverse images from somite 17 of each embryo. Cells were counted in original ZEN stacks and allocated to regions (Fig. 2A) in confocal stacks of epaxial somites of wholemount fish by scanning through in the XZ direction while toggling channels. Xanthophores were excluded from Pax7 counts based on nuclear shape, location and intensity (Hammond et al., 2007).

### 2.4 Statistics

Statistics were analysed with Microsoft Excel and AnalySoft Statplus, Graphpad Prism 6 or SPSS on the number of samples indicated. F-test was used to determine equivalence of variance and the appropriate Student's *t*-test or ANOVA with Scheffé post hoc test applied unless otherwise stated. All graphs show mean and standard error of the mean. Numbers on columns represent number of fish scored.

## 3 Results

### 3.1 Growth of zebrafish muscle

**Fig. 1:**
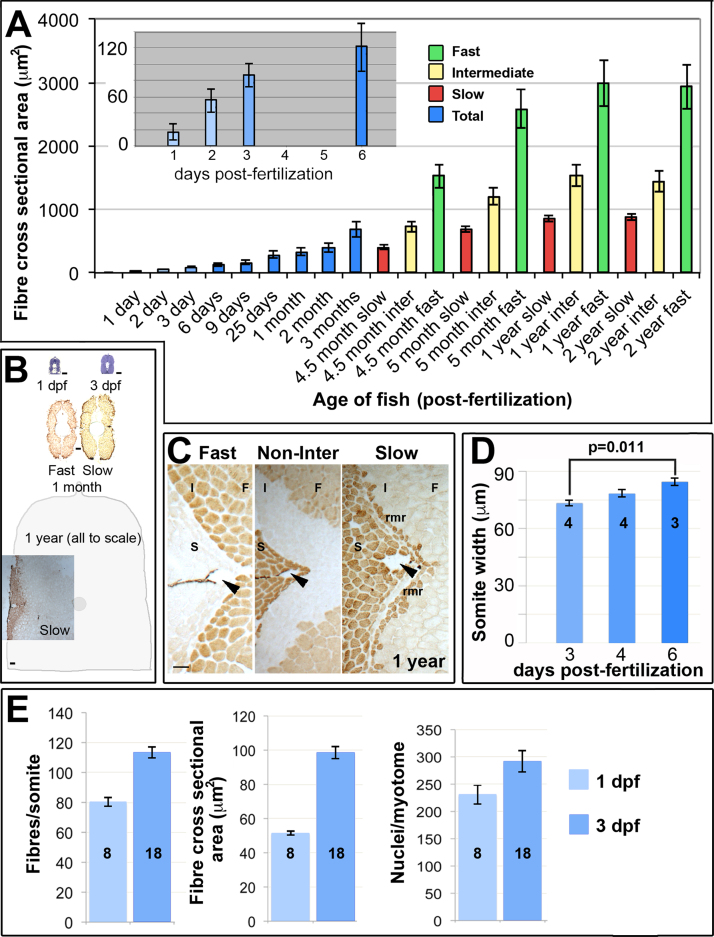
Muscle growth involves increase in fibre number and size. A. Fibre cross sectional area from unfixed cryosections as a function of age and fibre type in single average-sized fish at each age. Mean±SEM from 80 to 250 fast fibres and all slow and intermediate fibres in midbody somites. Inset magnifies early stages. B. Toluidine blue and myosin stained midbody sections at same scale Schematic indicates approximate size of entire section at 1 year. C. Immunodetection distinguishes fast (F), intermediate (I) and slow (S) fibres including the red muscle rim (rmr) from 4.5 months. Arrowheads indicate the lateral line. D. Mediolateral width of somite measured at horizontal myoseptum from wholemount confocal stacks. E. Fibres and nuclei were counted and cross sectional area measured on *YZ* confocal sections of somite 16–20 from 8 and 18 lightly-fixed Hoechst-stained *Tg(Ola.Actb:Hsa.HRAS-EGFP)* embryos at 1 and 3 dpf, respectively. As small fibres are hard to count with confidence in fixed preparations, fibre numbers represent minimal estimates. Note that the 1 dpf values for fibre cross sectional area are significantly larger in wholemount measurements (E) compared to cryosectioned material (A), possibly due to methodological and/or lay-to-lay variation. Bars 50 µm.

Muscle fibre cross sectional area was determined in embryonic, larval and adult zebrafish (Fig. 1). Mean fibre size increased dramatically in the embryonic period, less rapidly during larval life, slowly beyond 5 months and appeared to plateau after 1 year of age (Fig. 1A,B). In adults, fibre types were distinguished by myosin heavy chain (MyHC) content (Fig. 1B,C). As reported previously (Patterson et al., 2008), slow fibres were smaller than the adjacent intermediate fibres, with the more numerous fast fibres in the deep myotome being the largest (Fig. 1A). Paralleling the rapid increase in fibre size from 1 to 5 dpf, somites increase in mediolateral width (Fig. 1D; p = 0.011). Growth also involves increase in fibre number (Fig. 1E). The smallest fibres in developing somites are located near the DM, particularly at the epaxial and hypaxial somitic extremes, suggesting that new fibres arise from DM cells (Stellabotte et al., 2007). Although fibre number increases between 1 and 3 dpf (Fig. 1E and see below), mean fibre size doubles, despite the lowering effect on mean fibre size of small newly-added fibres (Fig. 1A,E). As no more than five new slow fibres are formed between 1 and 3 dpf, the remaining new fibres must be fast, in agreement with previous results (Barresi et al., 2001, Windner et al., 2012). Counts of nuclei within the myotome also show a 20% increase (Fig. 1E). The increase in myotome nuclei is sufficient to yield five mononucleate slow fibres and twenty extra fast fibres, but does not double like fibre size (Fig. 1E). As shown below, these trends continued until at least 6 dpf. Thus, both increase in fibre volume per nucleus and addition of nuclei to fibres by precursor myoblasts contribute to myotome growth.

### 3.2 Increase in Pax7^+^ cells parallels growth of early larval muscle

**Fig. 2:**
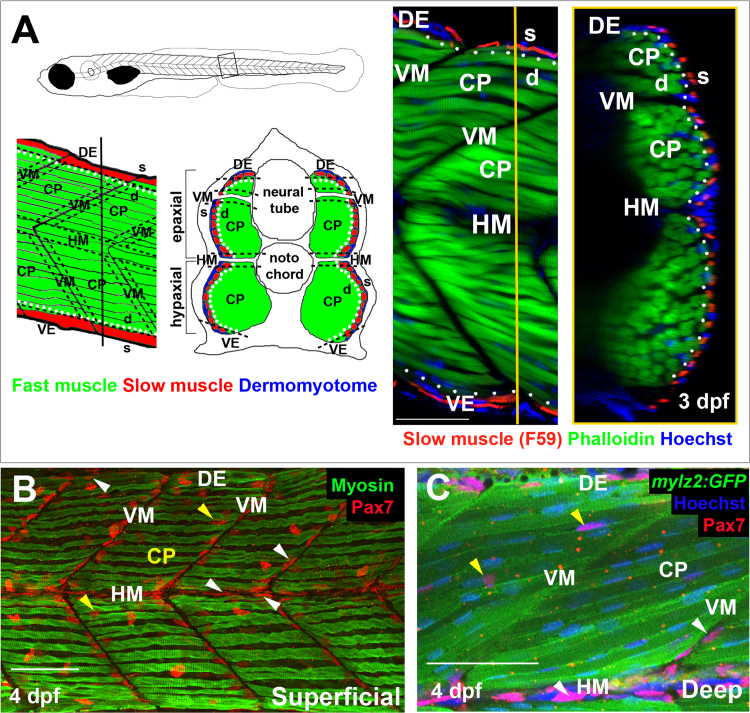
Spatial distribution of Pax7^+^ cells in growing myotome. A. Somites at the trunk/tail border shown schematically to define myotomal regions. Schematics (left panels) and confocal optical sections (right panels) of 3 dpf *Tg[mylz2:GFP]* (green) fish showing slow myosin (red) and nuclei (blue) in lateral (left; anterior to left, dorsal top) and transverse (right; dorsal to top, medial left) views. Epaxial (dorsal) and hypaxial (ventral) somite was conceptually segmented first into superficial (s; dermomyotome and underlying slow fibre layer, judged as about one nuclear length (~6 µm) from the peri/epidermal surface) and deep (d; fast myotomal and myosepta) domains (separated by dots). Within each domain, a central portion (CP) was distinguished from myoseptal border regions lying within a nuclear length (~6 µm) of the somite surface (indicated by dashes). Vertical myoseptum (VM; at which fibre ends from adjacent somites meet), horizontal myoseptum (HM; at which muscle pioneer cells and the lateral line separate epaxial and hypaxial somite domains) and dorsal and ventral edges of the DM (DE/VE; at which the lateral and medial myotome surfaces meet and nascent fibres form (Barresi et al., 2001, Johnston et al., 2009) were distinguished. **B,C.** Single confocal slices from wholemount 4 dpf larvae taken in lateral view, orientated with dorsal to top and anterior to left. Wt (B) or *Tg(−2.2mylz2:GFP)*^*i135*^ (C) larvae stained with anti-Pax7, Hoechst 33342 (detecting nuclei) and either A4.1025 (B, detecting sarcomeric MyHC) or anti-GFP (C). The superficial monolayer of slow muscle fibres aligned parallel to the horizontal myoseptum (HM) in somites 15–18 (B). Pax7^+^ nuclei surround the myotome (white arrowheads) at dorsal edge (DE), HM and vertical myoseptum (VM) and also occur in central portion (CP; yellow arrowheads) in both the epaxial and hypaxial domains. Pax7^+^ cells nestle amongst deeper fast fibres orientated oblique to HM in the CP region of the epaxial myotome (C). Bars 50 µm.

**Fig. 3:**
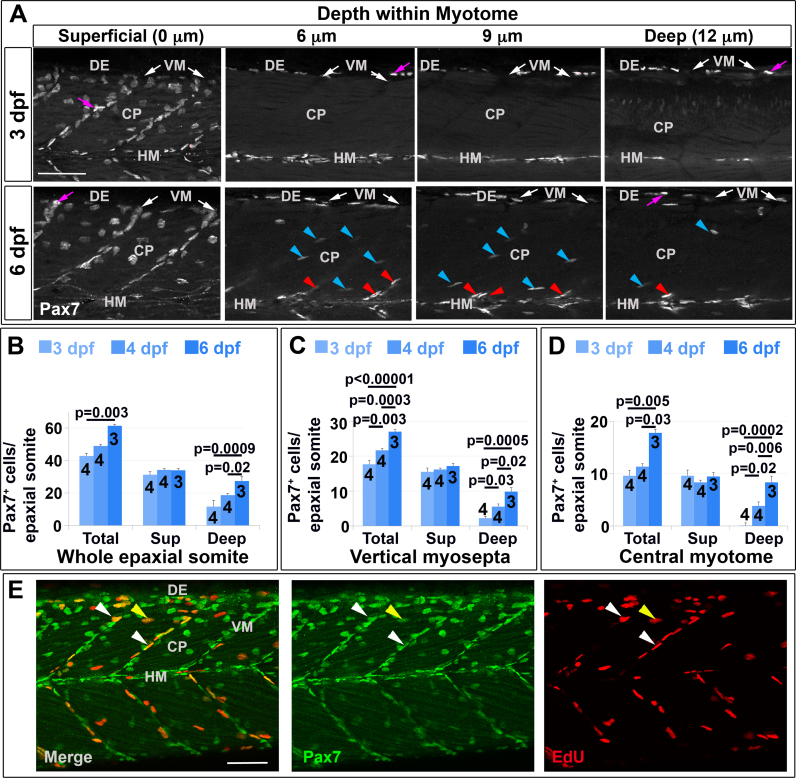
Pax7^+^ nuclei increase in deep myotome. Pax7 (A-E) and EdU (E) labelling in wholemount wt larvae. Single confocal slices of zebrafish larvae in lateral view. A: Flattened dehydrated embryos imaged at the indicated depths (full larval thickness approximately 35 µm). At 3 dpf, MPCs accumulate superficially near VM (white arrows) but are absent deeper within myotome. Xanthophores (purple arrows) are rare and bright. By 6 dpf, Pax7^+^ nuclei appear deep at the VM (red arrowheads) and CP (blue arrowheads). B-D**:** Numbers of Pax7^+^ nuclei in epaxial somites 16–18 of whole mount larvae increase with age. Mean±S.E.M. The small error bars indicate tight regulation of Pax7^+^ cell numbers. Number of embryos scored is indicated within the columns. E: Co-localization of Pax7 and EdU in MPCs of 4 dpf larva both at VM (white arrowheads) and CP (yellow arrowheads). Vertical myoseptum (VM), central portion (CP), horizontal myoseptum (HM), dorsal edge (DE). Bars 50 µm.

**Fig. S1:**
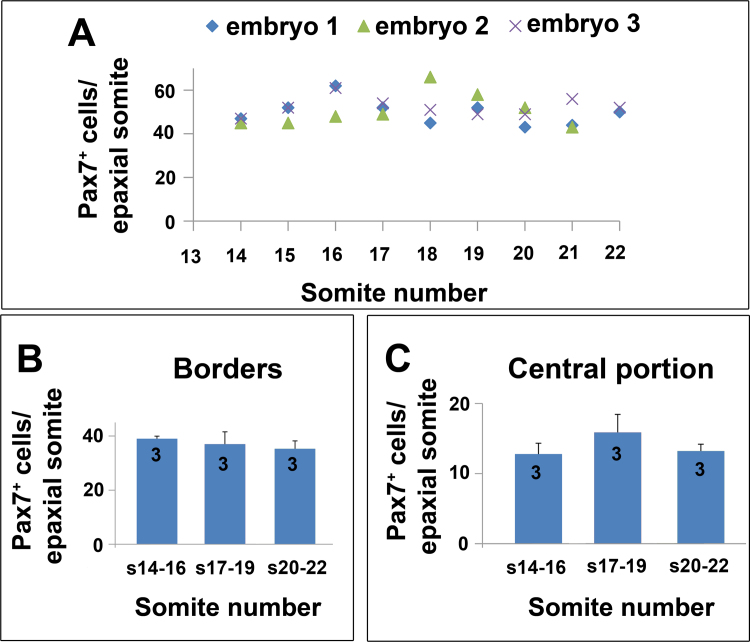
Low variance in the number of Pax7^+^ cells between larvae and somites. The number of Pax7^+^ cells in epaxial somites 14–22 of three 4 dpf zebrafish larvae were counted irrespective of depth. A. The total number of Pax7^+^ cells varied within a similar range between somites 14–22 in individual larvae. B. The number of Pax7^+^ cells in the somite borders did not differ significantly between the somite groups s14-16 and s17-19 [p = 0.35] and between s17-19 and s20-22 [p = 0.38]. C. The number of Pax7^+^ cells at central portion was also similar between the somite groups s14-16 and s20-22 [p = 0.39] and between s17-19 and s20-22 [p = 0.77]. Error bars are standard error of the mean.

From where do the extra nuclei and fibres derive? Pax3/7 proteins mark most dermomyotomal MPCs in both amniotes and zebrafish (Devoto et al., 2006, Relaix and Zammit, 2012). As no antibodies specific to zebrafish Pax3 are available, we focused on Pax7. Pax7^+^ cells in somites of developing 1 dpf zebrafish are located in a superficial domain lateral to differentiated muscle in the DM (Devoto et al., 2006, Feng et al., 2006, Hollway et al., 2007; see Fig. 2A for definition of somitic zones). New fibres originate in the superficial domain at the dorsal edge (DE) and ventral edge (VE) of the myotome (Barresi et al., 2001), where many Pax7^+^ cells are located (Fig. 2B,C). As somites mature, Pax7^+^ nuclei accumulate at the vertical and horizontal myosepta (Fig. 2B,C). Pax7^+^ cells at the vertical myoseptum (VM) are initially superficial, near the peri/epidermis, whereas those at the horizontal myoseptum (HM) can be deep within the somite (Fig. 2C). By 4 dpf, small numbers of Pax7^+^ cells are also observed deep within the central portion (CP) of both epaxial and hypaxial myotomes (Fig. 2C). As particular regions of the amniote dermomyotome give rise to distinct MPCs (Buckingham and Rigby, 2014), we analysed the changing numbers of Pax7^+^ cells in defined somitic zones (Fig. 2A). Immunolabelling of Pax7 in larvae prior to 4 dpf revealed that most Pax7^+^ cells were located at myotome borders (including dorsal edge DE, horizontal myoseptum HM, and vertical myosepta VM), with the remainder in the superficial central portion (CP), the central DM (Fig. 2, Fig. 3A). Subsequently, Pax7^+^ cells appeared deep within the myotome (Fig. 3A). As no temporal differences in Pax7^+^ cell behaviour in the epaxial and hypaxial somite were noted at any stage examined, and as numbers of Pax7^+^ cells per epaxial somite did not vary detectably along the rostrocaudal axis from somites 14–22 (Fig. S1), we chose to explore changes in Pax7^+^ cell number in the epaxial domain of somites 15–20. Between 3 and 6 dpf, the total number of Pax7^+^ cells per epaxial somite increased by about 50%, from ~40 to ~60 cells (Fig. 2B; p = 0.003). Strikingly, Pax7^+^ cell numbers did not change significantly in the superficial DM; the increase in Pax7^+^ nuclei was accounted for by a rise deep within the somite (Fig. 3A,B; p<0.001).

**Fig. S2:**
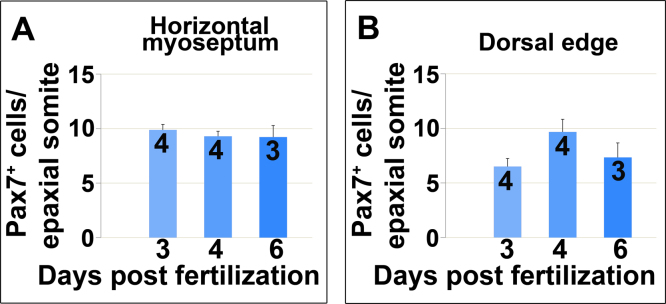
Quantitation of the number of Pax7^+^ cells at horizontal myoseptum and dorsal edge. Mean count of Pax7+ cells in horizontal myoseptum (A) and dorsal edge (B) domains of somites 16–18 in the number of fish indicated on the columns. No difference was significant.

To understand how Pax7^+^ cells arise in the deep myotome, the locations of Pax7^+^ cells were characterized at successive stages. In 3 dpf larvae, about half the Pax7^+^ cells were at the superficial vertical myoseptum (VM), mostly oriented with their long axes parallel to the vertical myoseptum (VM) (Fig. 3A-C). Some Pax7^+^ cells were in the superficial central portion (CP) and dorsal edge (DE) regions, and a few were located deep within the myotome at the horizontal myoseptum (HM) (Fig. 3A,C,D). By 6 dpf, in contrast, Pax7^+^ cell numbers had risen significantly in the deep central portion (CP), the central myotome, where the cells were aligned between muscle fibres (Figs. 2C, 3D; p<0.001) and within the deep vertical myoseptum (VM) (Figs. 2C, 3C, p<0.001). The long axes of some Pax7^+^ nuclei in deep vertical myoseptum (VM) were not parallel to the vertical myoseptum (VM), but pointed into the myotome, suggesting these cells may move between the vertical myoseptum (VM) and central portion (CP). Although the number of Pax7^+^ cells increased significantly in the deep VM and CP by 6 dpf, no change was detected in superficial vertical myoseptum (VM) or central portion (CP) (Fig. 3C, D; p = 0.302 and 0.942, respectively). Further, the number of Pax7^+^ cells in horizontal myosepta (HM) and at the dorsal edge (DE) was unchanged from 3 to 6 dpf (Fig. S2). These data show that the number of Pax7^+^ cells increases at specific somitic locations during larval muscle growth and their orientation is suggestive of an inward movement.

**Fig. S3:**
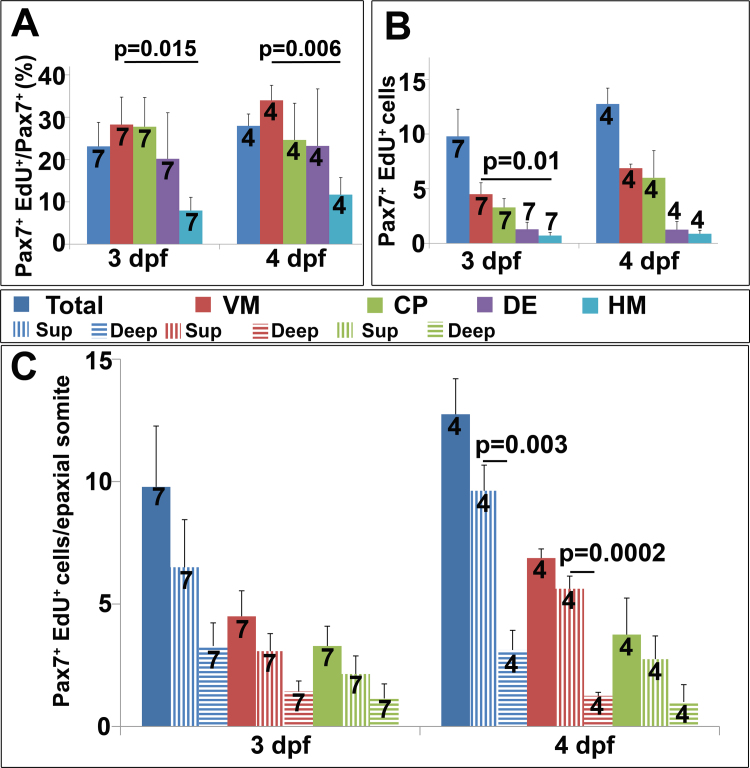
Location of Pax7^+^ cell proliferation. A: The fraction of Pax7^+^ cells labelled with EdU is similar in most somitic domains. B: Paralleling the higher Pax7^+^ cell numbers, slightly more proliferative Pax7^+^ cells were detected at VM than HM (compare values with Fig. 3D and S2A). C: Pax7^+^ cells in both deep and superficial VM and CP proliferate similarly (compare values with Fig. 3C,D).

The zebrafish DM contains proliferating Pax7^+^ cells (Hammond et al., 2007, Stellabotte et al., 2007), which could act as a source of cells entering the deep myotome. Analysis of Pax7^+^ cell proliferation with a 3 h EdU labelling pulse showed that around 20% Pax7^+^ cells are in S-phase in most somitic regions at 3 and 4 dpf, when cells are beginning to enter the deep myotome (Fig. 3E and S3). These findings extend those of Barresi et al. (2001), who reported that around 20% of total nuclei in the dorsal region of somite were labelled at 96 hpf by a 1 h BrdU pulse and most were on the somite surface. Proliferation therefore contributes to the increase in Pax7^+^ cells, but the increase in Pax7^+^ cells in the deep myotome does not arise from localised proliferation.

### 3.3 Pax7^+^ cells migrate into the myotome and proliferate

**Fig. S4:**
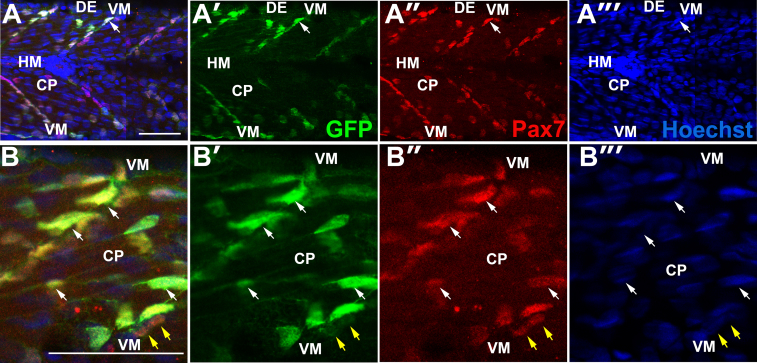
*pax7a:GFP*^*+*^ cells co-express Pax7. Wholemount 3.25 dpf *pax7a:GFP*;*pfe/pfe* larvae in lateral view with dorsal up and anterior to left after immunodetection of GFP and Pax7, and counterstaining with Hoechst 33342. Single deep confocal slices of two whole somites (A) and epaxial somite (B) are shown. GFP largely co-localizes with Pax7 (white arrows). Pax7^+^ cell on VM that is not GFP^+^ (yellow arrows). Scale bars = 50 µm.

In amniotes, Pax7^+^ cells of the dermomyotome have been shown to enter the myotome when the central dermomyotome disperses (Ben-Yair and Kalcheim, 2005, Gros et al., 2005, Kassar-Duchossoy et al., 2005). To visualize the dynamics of Pax7^+^ cells in live growing fish, a *pax7a* reporter transgene *Tg(pax7a:EGFP)*^t32239Tg^ was bred onto a *pfeffer*^*tm236b*^ background to remove xanthophores, which would otherwise express Pax7 (Alsheimer, 2012, Mahalwar et al., 2014, Minchin and Hughes, 2008, Odenthal et al., 1996). *Pax7a:*GFP^+^ cells and Pax7^+^ nuclei were largely co-localised at 3.25 dpf (Fig. S4), and GFP^+^ cells form muscle fibres, consistent with our findings in the regeneration context (Knappe et al., 2015, Pipalia et al., 2016).

**Fig. 4:**
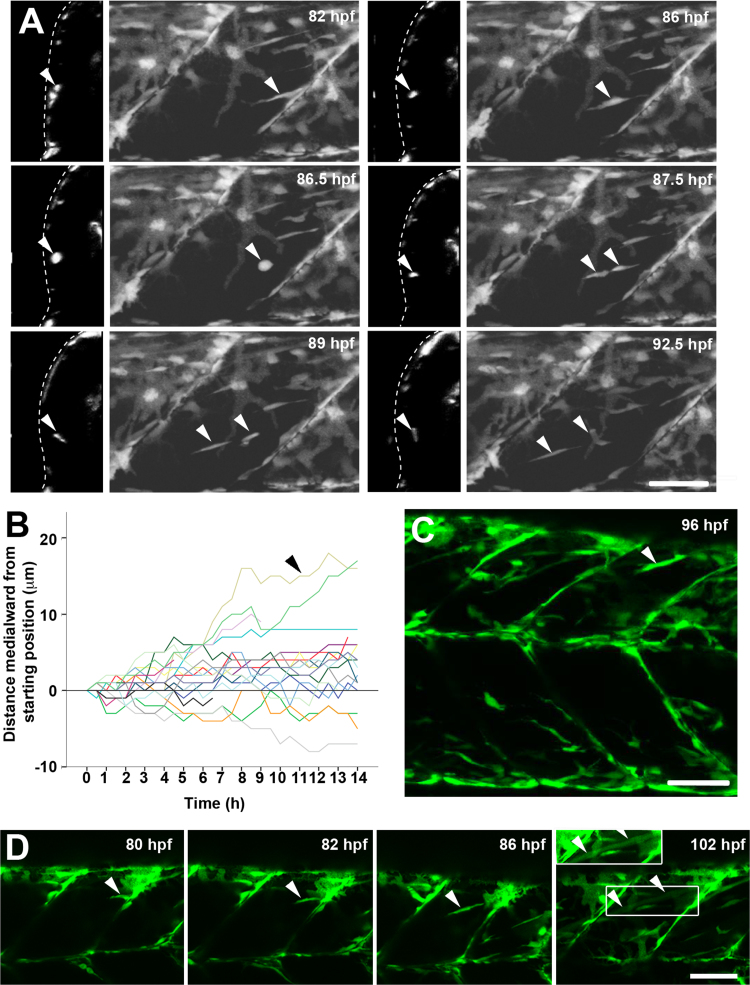
p*ax7a:GFP*^*+*^ cells migrate into deep central myotome and differentiate. Confocal maximum intensity *Z* projections and orthogonal *YZ* views of live *pax7a:GFP*;*pfe/pfe* larvae in lateral view. A. Timelapse of 3.5–4 dpf larva taken every 30 min for 14 h showing migration of a cell from the posterior vertical myoseptum into the deep myotome (arrowheads). Note rounding up, division and separation of daughters. B. Analysis of distance moved towards midline in *Z* plane for each cell in the epaxial somite from the timelapse shown in A. Note that total movement of cells is often greater, as migration in the anteroposterior and dorsoventral planes (*XY*) is not shown. Each cell was measured relative to its starting position. Arrowhead indicates the cell highlighted in A. C. A cell extending from the dorsal edge into the deep myotome (arrowhead). D. Timelapse of myotube formation from a cell entering the myotome from the posterior VM (arrowheads). Between 86 and 102 hpf the GFP became diluted in the extended fibre (box is shown with contrast enhancement in inset above). Bars 50 µm.

Time-lapse confocal analysis of live *pax7a:GFP;pfe*^*tm236b/tm236b*^ larvae showed many *pax7a:GFP*^+^ cells oriented parallel to the vertical myosepta (VM) at 3.5 dpf (Fig. 4A). Occasional VM cells extended into the myotome parallel to adjacent fast fibres, migrated into the deep myotome and some subsequently divided (Fig. 4A). Tracking of all GFP^+^ cells in one epaxial myotome from 3.5 to 4 dpf revealed that while a few cells moved medially up to 20% of somite width, most moved little (Fig. 4B). Nevertheless, rather few were carried away from the midline, despite the thickening of the myotome (compare Fig. 1D and 4B). GFP^+^ cells were observed to enter the myotome from vertical myosepta (VM) and dorsal edge (DE) (Fig. 4C, Movie S1), but not directly from the central region of the DM. Few GFP^+^ cells were observed deep in the somite at 3 dpf, but such cells were readily detected from 4 dpf onwards (Fig. 4). We conclude that proliferation and migration of cells from the VM and DE contribute to the rise in Pax7^+^ cells within the deep CP. As the number of Pax7^+^ cells in the superficial somite and vertical myosepta (VM) is undiminished over the period studied, we suggest that proliferation of Pax7^+^ cells is sufficient to replenish the loss of cells from these pools following their migration into the deep CP.

Supplementary material related to this article can be found online at doi:10.1016/j.ydbio.2017.08.029.

The following is Supplementary material related to this article Movie 1.

Movie 1 Migration of *pax7a:GFP*^*+*^ cell into myotome. Time lapse confocal movie showing migration of a single *pax7a:GFP*^*+*^ MPC from the VM close to DE into the myotome. The same cell was tracked through three successive planes (level indicated at lower left) over a 5.5 h time period (time indicated at upper left). The video starts with a superficial plane, showing the cells disappear during the first 3 h. The 6 µm plane starts at t = 0 and shows the cell appearing and continuing deeper into the somite over the first 3 h. The 13 µm plane also starts at t = 0 with no cell visible and shows the appearance and extension of a long initial cell protrusion followed by the cell body.

### 3.4 Pax7^+^ cells make muscle

**Fig. 5:**
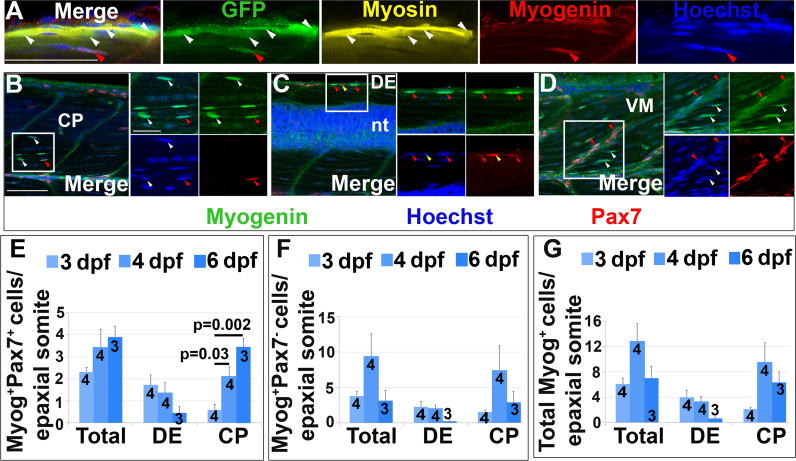
Pax7^+^ cells differentiate in specific somite regions. Single confocal planes of 4 dpf wholemount immunofluorescence in lateral view. Scale bars 50 µm. A. *pax7a:GFP*;*pfe/pfe* larva showing GFP in a MyHC^+^ muscle fibre (white arrowheads). A deep GFP^+^MyHC^-^ cell co-labels with Myogenin (red arrowheads). B-D. Pax7 and Myogenin in epaxial somite of wt larva showing Pax7^+^Myog^+^ cells (red arrowheads) in CP (B), DE (C) and VM (D), Pax7^+^Myog^-^ cells (yellow arrowheads) in DE (C) and Pax7^-^Myog^+^ cells (white arrowheads) in CP (B,D). Note the reduced Pax7 and Myog signal in Pax7^+^Myog^+^ cells. E-G. Time course and location of Myog^+^Pax7^+^ (E), Myog^+^Pax7^-^ (F) and total Myog^+^ (G) cells.

Pax7^+^ MPCs give rise to muscle fibres in amniotes (Kassar-Duchossoy et al., 2005, Lepper and Fan, 2010). Co-expression of Pax7 and Myogenin in zebrafish suggests a similar progression (Devoto et al., 2006). Neither in fish nor amniotes, however, has Pax7 mRNA or protein been reported in fibres themselves. Time-lapse analysis of *pax7a:GFP* fish showed that Pax7^+^ cells occasionally formed fibres with weak GFP (Fig. 4D) consistent with our previous work (Pipalia et al., 2016). More sensitive immunodetection revealed elongated GFP^+^ fibre-like structures containing sarcomeric MyHC at 4 dpf (Fig. 5A). Thus, perdurant GFP proved that Pax7^+^ cells contribute to muscle growth.

Some GFP^+^ mononucleate cells in the myotome were found to contain Myogenin (Fig. 5A), a marker of differentiating myoblasts in amniotes and embryonic zebrafish (Hasty et al., 1993, Hinits et al., 2009, Nabeshima et al., 1993). Dual immunodetection of Myog and Pax7 proteins in epaxial somites between 3 and 6 dpf confirmed Pax7^+^Myog^+^ cells in central portion (CP), dorsal edge (DE) and more rarely in vertical myosepta (VM) (Fig. 5B-D, respectively). Similarly, Pax7^-^Myog^+^ cells were predominantly in the central portion (CP), occasionally at the dorsal edge (DE), rarely at horizontal myoseptum (HM) and were not observed at vertical myosepta (VM) (Fig. 5B-D and data not shown). Pax7^+^Myog^+^ cells tended to show weaker Pax7 and Myog labelling than in the respective single-positive cells (Fig. 5B-D), suggesting a transition between Pax7^+^Myog^-^ and Pax7^-^Myog^+^ cells. The predominant locations of Myog^+^ cells in central portion (CP) and dorsal edge (DE) suggest these are the major regions of myoblast terminal differentiation.

Counting Myog^+^ cells revealed a transition in muscle differentiation between 3 and 6 dpf. At 3 dpf, most Myog^+^ cells were located in the dorsal edge (DE). From 4 dpf onwards, most Myog^+^ cells were in the central portion (CP) (Fig. 5E-G). The total number of Pax7^+^Myog^+^ cells increased between 3 and 6 dpf (p = 0.02, Fig. 5E), primarily due to an increase in Pax7^+^Myog^+^ cells in the central portion (CP) (p = 0.002); there was no change at the dorsal edge (DE) (p = 0.08). These results indicate that many of the Pax7^+^ cells that invade the central myotome rapidly differentiate and contribute to myotome growth. We conclude that although terminal differentiation removes Pax7^+^ cells, proliferation and migration is sufficient to replenish the Pax7^+^ cell pool.

### 3.5 Altered growth in *myod^fh261^* mutants

**Fig. 6:**
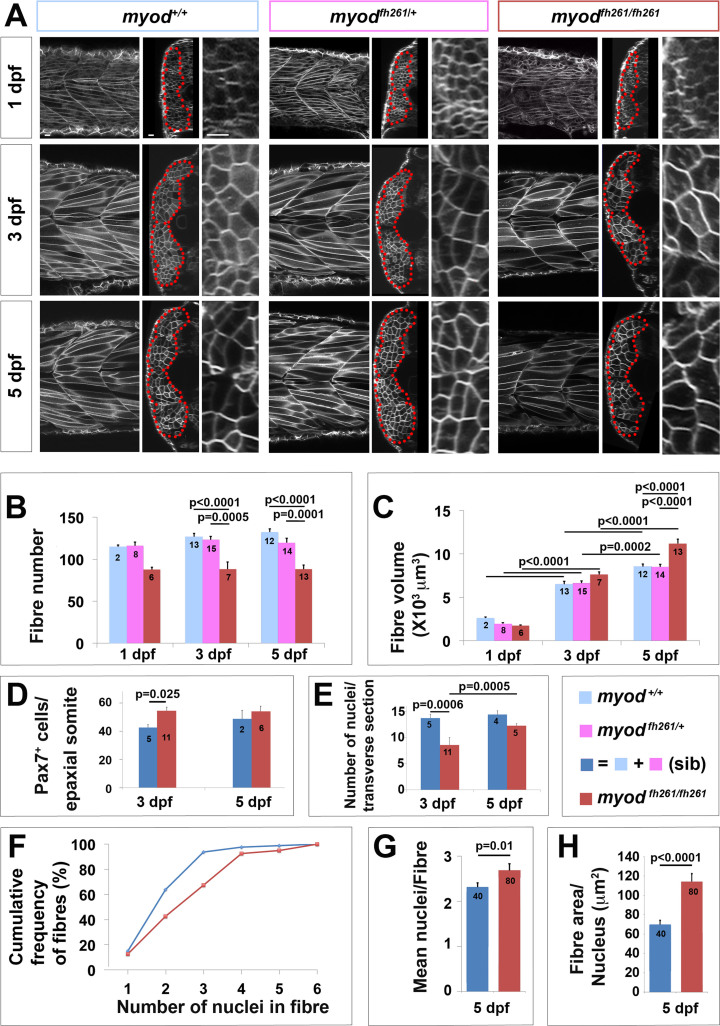
Lack of *myod* alters somite growth. A. Single confocal planes of *myod*^*fh261*^ mutant and sibling zebrafish expressing plasma membrane-GFP from *Tg(Ola.Actb:Hsa.HRAS-EGFP)*^*vu119*^. Individual retrospectively-genotyped larvae are shown at comparable levels in somites 16–18 at successive stages in each tryptich in lateral (left), transverse (centre) and magnified transverse (right) views. The somite is outlined (red dots). Bars 10 µm. B. Fibre number per somite in each stage and genotype as indicated in key. Note that sibling fibre numbers at 1 dpf are higher than those in Fig. 1E, perhaps because sequential live scanning means that ‘1 dpf’ fish are on average ~4 h older than in fixed preparations. C. Mean fibre volume = myotome volume/fibre number. D,E. Larvae from *myod*^*fh261*^ heterozygote incross were stained at 3 and 5 dpf for Pax7, MyHC and DNA and analysed by confocal microscopy. Pax7^+^ cell number/epaxial somite (D) and number of nuclear profiles within the myotome/transverse optical section of epaxial somite 17 (E) were scored from fish genotyped by loss of head myogenesis. F-H. Analysis of nuclear number in fast fibres of *myod*^*fh261*^ mutant and sibling *Tg(Ola.Actb:Hsa.HRAS-EGFP)*^*vu119*^ injected with RNA encoding H2B-mCherry to permit counting in live larvae. Fibres analysed (number on columns) are shown in Fig. S5. A cumulative frequency plot (F) reveals the larger number of nuclei in fibres of mutants, which is reflected in an average of 16% increase in nuclear number (G) accompanied by a 63% increase in fibre cross-sectional area per nucleus (H), reflecting an 80% increase in fibre size. Differences tested by ANOVA with Tukey post-hoc (B,C), Kruskall-Wallis (F,G) and *t*-test (E,H).

Embryos lacking *myod* function show a 50% reduction in muscle at 1 dpf accompanied by a twofold excess of MPCs. This is followed by rapid growth resulting in recovery such that little if any myotome size difference remains by 5 dpf (Hammond et al., 2007, Hinits et al., 2011). Recovery did not, however, lead to normal muscle. Analysis of fibre number and size revealed that at 5 dpf *myod*^*fh261*^ mutants have 33% fewer fibres, but these are about 30% larger than those in wt (Fig. 6A-C). Thus, recovery compensated for the reduced fibre number by increased fibre growth.

Analysis of fibre number and size during muscle recovery showed significant defects in *myod*^*fh261*^ mutants. In wt embryos, fibre numbers increased by about 15% from 1 to 5 dpf. No increase was observed in *myod*^*fh261*^ mutants (Fig. 6B). In contrast, fibre size was comparable in wt and *myod*^*fh261*^ mutants at 1 dpf, but fibre size increased faster in mutants so that, by 5 dpf, fibres were larger, thereby compensating for the reduction in fibre number (Fig. 6C).

*Myod*^*fh261*^ mutants have an increased number of Pax3/7^+^ cells at 1 dpf, paralleling the reduction in muscle differentiation (Hinits et al., 2011). The number of Pax7^+^ cells remains elevated at 3 dpf, but returns almost to normal by 5 dpf, accompanied by a rise in the number of myonuclei in the myotome (Fig. 6D,E).

**Fig. S5:**
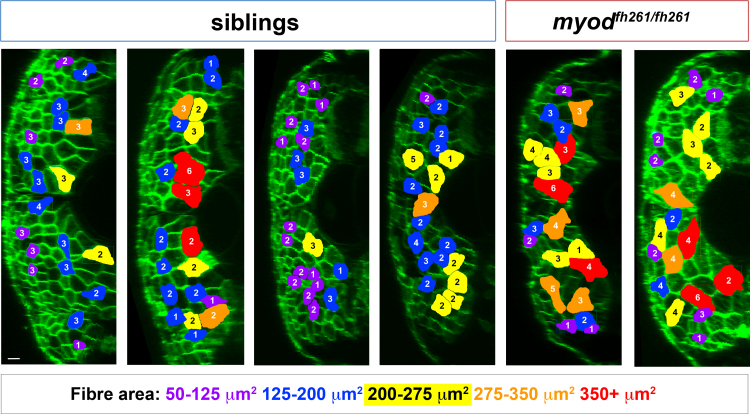
Nuclear counts and size measurement of fast fibres. Transverse views of wholemount 5 dpf larvae from an incross of *myod*^*fh261/+*^;*Tg(Ola.Actb:Hsa.HRAS-EGFP)*^*vu119*^ injected at 1 cell stage with RNA encoding H2B-mCherry (similar to Addgene #20972, red channel not shown). At 5 dpf, fully dual-labelled larvae were scanned in 3D confocal stacks and retrospectively genotyped. Coloured fibres indicate those selected for measurement using the criteria of a) unambiguously delineation of plasma membrane and nuclei and b) distribution throughout the fast myotome of somite 17. The cross-sectional area at the anteroposterior midpoint of each fibre was measured using Volocity v6.3 and the number of nuclei counted manually in twenty fibres in each fish. Note that fibre outline does not perfectly match the shape of fibre because the midpoints of most fibres are in different planes. Size of fibre is colour coded and nuclear number indicated on each fibre. Data for all sibling (80) and all mutant (40) fibres were pooled and presented in Fig. 6F-H. Scale bar = 10 µm.

Do *myod*^*fh261*^ mutants recover by fusion of the excess myoblasts into the pre-existing fibres? Both the maximal number and the mean number of nuclei in single fast muscle fibres of *myod*^*fh261*^ mutants was higher than in siblings (Fig. 6F,G and S5), suggesting that the excess myoblasts contribute to the growth of existing myotomal fibres. However, when the fibre size per nucleus was calculated (by dividing the cross-sectional area of each fibre by its nuclear number), *myod*^*fh261*^ mutants had a clear increase in effective nuclear domain size (Fig. 6H and Appendix A, Appendix A). Thus, fusion of excess Pax7^+^ DM cells into pre-existing fibres during recovery of *myod*^*fh261*^ mutants accompanies hypertrophy – an increase in fibre volume per nucleus.

### 3.6 Excess Pax7^+^ cells in the deep myotome of 3 day *myod^fh261^* mutants

**Fig. 7:**
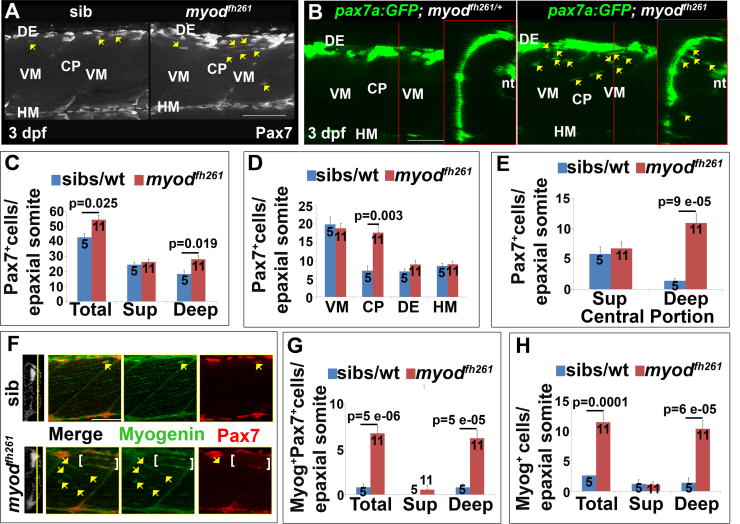
Premature ingression of Pax7^+^ myogenic precursors in *myod*^*fh261*^ mutants. Wholemount larvae from a *myod*^*fh261/+*^ incross stained for Pax7 (A,C-H) or *pax7a:GFP;myod*^*fh261/+*^ incross imaged live (B) at 3 dpf and shown in confocal short stacks in lateral view. A. Pax7 antibody stained nuclei (arrows) within the deep CP myotome. B. Pax7:GFP^+^ cells (arrows) in lateral and transversal views. C-H. Comparison of Pax7^+^ (C-E), Myog^+^Pax7^+^ (G) and Myog^+^ (H) cell numbers in the epaxial half of som16-18 between the number of *myod*^*fh261*^ mutants and their siblings indicated within columns. The extra Pax7^+^ cells in *myod*^*fh261*^ mutants (C) were specifically located in deep CP (D,E). Lateral planes at deep locations indicated by yellow line on transversal sections of wholemount larvae stained for Pax7, Myogenin and Hoechst (F) reveal increased numbers of Myog^+^ and Pax7^+^Myog^+^ in the deep CP (arrows). Note the alignment of some Myog^+^ nuclei (brackets). VM: vertical myosepta, CP: central portion, DE: dorsal edge, HM: horizontal myoseptum. Bars 50 µm.

To understand the contribution of DM cells to the recovery of *myod*^*fh261*^ mutants the number and location of Pax7^+^ and Myog^+^ cells were determined (Fig. 7). In 3 dpf *myod*^*fh261*^ mutants there were approximately 25% more Pax7^+^ cells than in wt (p = 0.025, Fig. 7A-C). Strikingly, the extra Pax7^+^ cells in mutants at 3 dpf were mostly located in the deep CP myotome (Fig. 7B-E). This reveals an earlier presence of Pax7^+^ cells in the deep myotome of *myod*^*fh261*^ mutants than in siblings and wt fish, which accumulate such cells from 4 dpf (Fig. 4).

**Fig. S6:**
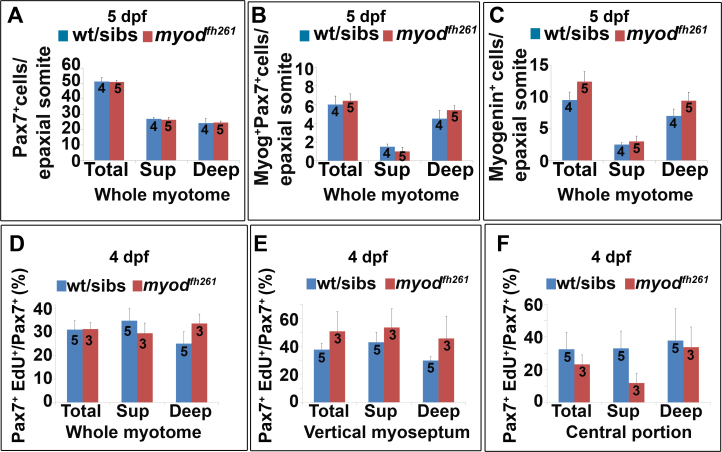
Proliferation and differentiation of MPCs in *myod*^*fh261*^ mutants and siblings. A-C. Wholemount *myod*^*fh261*^ mutants and siblings were stained for Pax7, Myogenin, Myosin and DNA at 5 dpf and numbers of Pax7^+^ (A), Pax7^+^Myog^+^ (B) and Myog^+^ (C) cells quantified in epaxial somite 17 from confocal stacks of the number of larvae indicated within columns. D-F. Wholemount EdU-treated *myod*^*fh261*^ mutants and sibling larvae were stained for EdU, Pax7, Myosin and DNA at 4 dpf and the fraction of Pax7^+^ cells marked with EdU was counted in confocal stacks of the whole epaxial somite 17 (D), the vertical myosepta (E) and the central portion (F).

The extra Pax7^+^ cells in 3 dpf *myod*^*fh261*^ mutants appear to be undergoing differentiation. More Myog^+^ cells were observed in the deep CP of *myod*^*fh261*^ mutants at 3 dpf, compared with their siblings (Fig. 7F-H). Moreover, these increases were observed specifically in the deep central myotome (Fig. 7G,H), and not at other locations of the myotome. Comparing *myod*^*fh261*^ mutants and siblings, a similar fraction of Pax7^+^ cells were also Myog^+^ and the ratio of Pax7^+^Myog^+^ to Pax7^-^Myog^+^ cells was unaltered between genotypes. These findings suggest that, once in the deep central myotome, *myod*^*fh261*^ mutant Pax7^+^ cells progress to terminal differentiation in the normal manner. At 5 dpf, no significant difference in either Pax7^+^ or Myog^+^ cell numbers persisted (Fig. S6A-C). These data argue that the premature appearance of Pax7^+^ cells in the deep central myotome does not reflect a failure of differentiation in *myod*^*fh261*^ mutant, but rather an adaptive process contributing to increase in nuclei/fibre and muscle mass recovery.

If the increase in MPCs in the deep central myotome reflects an adaptive process, it could arise either from increased migration or proliferation of Pax7^+^ cells. To examine this issue, *myod*^*fh261*^ was crossed onto the *pax7a:GFP* transgene to permit tracking of cell dynamics. Profiles suggesting migration of cells from the vertical myosepta into the deep myotome were more common at 3 dpf in *myod*^*fh261*^ mutants than in siblings (Fig. 7B and data not shown). Moreover, EdU labelling showed that cell proliferation was similar in the deep central myotome in mutants and siblings (Fig. S6D-F). We conclude that there is an increased migration of Pax7^+^ cells into the deep myotome of 3 dpf *myod*^*fh261*^ mutants.

### 3.7 Blockade of GSK3 reduces accumulation of Pax7 cells in the myotome

**Fig. 8:**
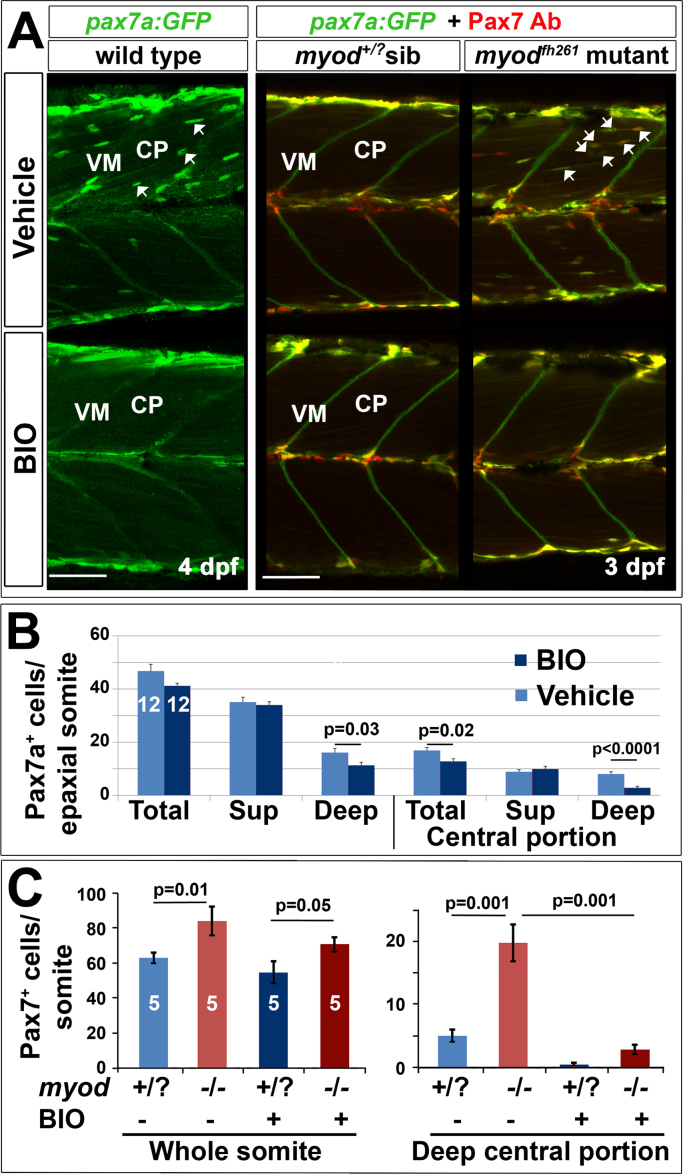
Blockade of ingression of Pax7a^+^ cells by BIO. The *pax7a:GFP* BAC transgene was bred onto a *pfe/pfe* background to diminish xanthophores (left panels). Such fish bred onto *myod*^*fh261/+*^ were in-crossed (right panels) and genotyped by sequencing. Larvae treated for 24 h with BIO or vehicle were stained in wholemount for GFP, Myog and DNA (left panels) or GFP, Pax7 (right panels). Confocal images are maximum intensity projections of short stacks in the deep myotome in lateral view with dorsal up and anterior to left, showing the decline of GFP^+^ cells (arrows) between fibres in the deep central myotome. VM vertical borders, CP central portion. Bars 50 µm. B. Number of pax7a:GFP^+^ cells in epaxial region of somites 16–18 of 12 BIO-treated 4 dpf embryos compared with 12 controls. Significantly fewer Pax7a^+^ cells were present deep within the somite, which was accounted for by highly significant loss from the deep CP. Sup = superficial. C. Larvae from an incross of *pax7a:GFP;myod*^*fh261/+*^ were sorted for GFP, treated at 2.25 dpf with BIO or vehicle, stained at 3.25 dpf for GFP, Pax7 and myosin and genotyped by head muscle. Total (left) and deep CP (right) Pax7^+^ cells were counted from confocal stacks of five larvae in each condition and differences tested by ANOVA with Bonferroni multiple comparison test.

**Fig. S7:**
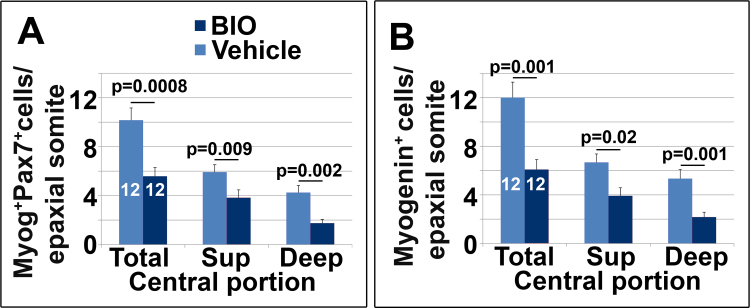
Reduction of MPC differentiation by BIO. The *pax7a:GFP* BAC transgene was bred onto a *pfe/pfe* background to diminish xanthophores and larvae at 3 dpf treated with BIO or vehicle were stained in wholemount at 4 dpf for GFP, Myog and DNA. The number of pax7a:GFP^+^Myog^+^ (A) and Myog^+^ (B) cells in the epaxial central portion of somites 16–18 of 12 BIO-treated embryos compared with 12 controls. Data are from the same experiment as shown in Fig. 8B. Sup = superficial.

A small molecule screen for pathways that affect Pax7^+^ cell behaviour revealed that GSK3 signalling may regulate migration (Fig. 8). GSK3 is downstream of various signalling pathways, including Wnt/β-catenin and Insulin, both of which can influence muscle growth (Bentzinger et al., 2014, Fernandez et al., 2002, Jones et al., 2015, Musaro et al., 2001, Tee et al., 2009). Treatment of wt larvae at 3 dpf with the GSK3 antagonist (2′Z,3′E)−6-bromoindirubin-3′-oxime (BIO) for 24 h reduced the number of Pax7^+^ cells in the deep CP myotome compared with vehicle (Fig. 8A). Quantification revealed that the number of GFP^+^ cells was significantly decreased in the deep CP of BIO-treated larvae, but relatively unaffected elsewhere (Fig. 8B). The numbers of differentiating Pax7^+^ cells and Myog^+^ cells were also significantly reduced in both superficial and deep CP (Fig. S7). Importantly, BIO also blocked the premature entry of Pax7^+^ cells into the myotome in *myod*^*fh261*^ mutants (Fig. 8C). Thus, BIO prevents migration and/or accumulation of Pax7a^+^ cells in the deep CP and alters terminal differentiation.

### 3.8 BIO fails to reduce compensatory muscle fibre growth

**Fig. 9:**
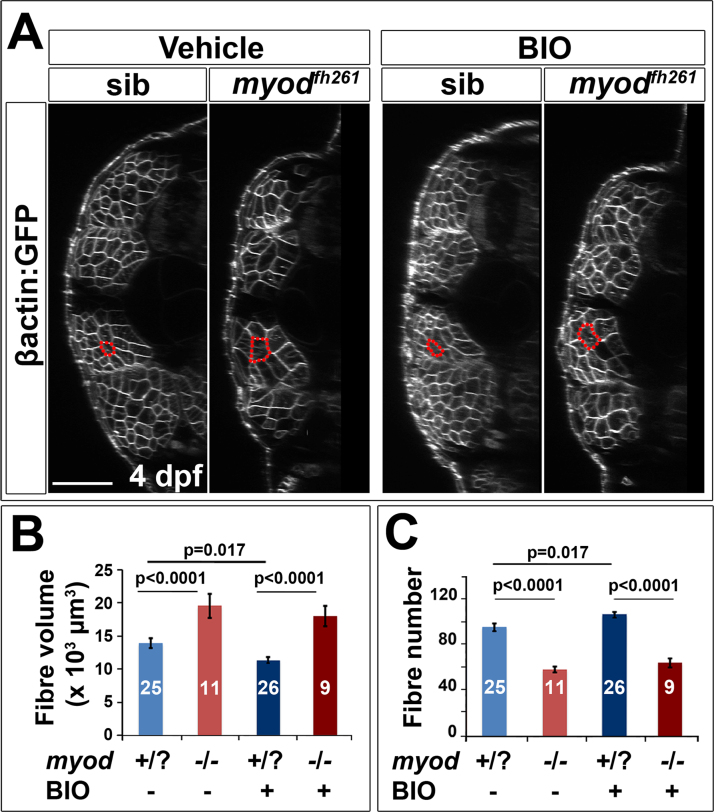
BIO does not prevent fibre hypertrophy in *myod*^*fh261*^ mutant. A. Larvae from an incross of *myod*^*fh261/+*^;*Tg(Ola.Actb:Hsa.HRAS-EGFP)*^*vu119*^ were sorted for GFP, treated with BIO or vehicle from between 2 and 3 dpf and imaged live at 4 dpf by confocal microscopy. Red outlines highlight typical fast fibres. Bar 50 µm. B,C. Quantification of mean fibre volume (B) and fibre number (C) from two replicate experiments was performed blind. Each larva was retrospectively sequence genotyped and the data pooled. Differences were tested by ANOVA with Bonferroni multiple comparison test.

As GSK3 inhibition led to fewer Pax7^+^ cells migrating into the deep myotome, it was possible to investigate the importance of this migration in recovery of the muscle in *myod*^*fh261*^ mutants. When *myod*^*fh261*^ mutant embryos were treated with BIO at 2 dpf, thus blocking the premature ingression of Pax7^+^ cells to the deep CP, the increase in muscle fibre volume triggered by the loss of Myod still occurred (Fig. 9A,B). Interestingly, BIO caused a slight rise in fibre number in wt siblings, but in *myod*^*fh261*^ mutants there was no significant change in fibre number (Fig. 9C). These data indicate that hypertrophy of fibres by increase in volume per nucleus provides the robust recovery of muscle in *myod*^*fh261*^ mutants and migration of Pax7^+^ cell from DM is not required for this recovery.

## 4 Discussion

The present work demonstrates that Pax7^+^ MPCs contribute to larval muscle growth and contains four major findings relevant to myogenesis. First, we show that Myod function is required for formation of the correct number of fast muscle fibres. Second, we describe regional variation in the behaviour of MPCs that likely reflect local control of their proliferation and subsequent differentiation during development. Third, we demonstrate a role for GSK3 signalling in regulating MPC migration, and possibly differentiation, within the early somite. Fourth, we find that muscle stem and precursor cell populations in the early somite are precisely regulated in response to perturbations so as to return the system rapidly to near-normal status, yielding developmental robustness. Our observations suggest that much of the control of myogenesis, even in a simple system, remains unexplained and advance the zebrafish myotome as a single cell-resolution vertebrate model for the quantitative time-lapse study of tissue growth and maintenance.

### 4.1 Regulation of fibre number

Fish lacking Myod have one third fewer somitic fast muscle fibres than siblings carrying a wild type *myod* allele. Initially, *myod*^*fh261*^ mutants also have an excess of MPCs, suggesting that reduced differentiation explains the reduced fibre number. Although mutants recover muscle volume through fibre hypertrophy, fast fibre number does not recover. Despite the early reduction in total myonuclei in *myod*^*fh261*^ mutants, they show partial recovery by 5 dpf. Thus, the recovery of myotome volume previously reported in *myod*^*fh261*^ mutants (Hinits et al., 2011) involves both hypertrophy and elevated fusion of MPCs, leading to more nuclei in the enlarged fast fibres. Although some excess MPCs differentiate, they fail to make new fibres. This argues for a critical window in myotomal fast muscle development during which the first fibre cohort is initiated and the number of its fibres determined.

In fish, a prolonged initial period of stratified hyperplasia, in which new fibres are made at the dorsal and ventral edges of the myotome, leads to myotome growth (Rowlerson and Veggetti, 2001). This is followed, in many species, by mosaic hyperplasia, in which new fibres are formed between existing fibres in a manner similar to amniote secondary fibre formation. Environmental changes that alter fish behaviour also affect fibre formation (Johnston et al., 2009, Johnston et al., 2003, Macqueen et al., 2008), but it is unclear whether either stratified or mosaic hyperplasia is nerve-dependent (Johnston et al., 2011). One study that examined the relationship of initial fibre number and final muscle mass revealed that final fibre number is under genetic selection, but did not determine at what point in development the regulatory genes act (Johnston et al., 2004). We observed a failure of initial fibre formation in *myod*^*fh261*^ mutant larvae and a failure to recover from the initial deficit despite the presence of many Pax7^+^ MPCs. This finding contrasts with the situation in *tbx6* mutant fish, in which a transient delay in new fast fibre formation between 1 and 2 dpf recovers by 3 dpf in spite of a transient local reduction in Pax7^+^ MPCs (Windner et al., 2012). Our finding that, in the absence of new fibre formation, a gradient of fibre sizes persists within the fast myotome suggests that stratified hyperplasia is not the sole cause of such fibre size gradients. Future studies in larval zebrafish, where the limited MPC and fibre numbers make quantification practicable, may provide deeper insight into the control of fibre number.

### 4.2 Regional variations in division, migration and differentiation of MPCs

MPCs in wt fish behave distinctly depending on their somitic location. Each epaxial somite has about 40 Pax7^+^ MPCs at 3 dpf, a number that correlates well with the number of nuclei present in the somite that are not part of fibres (S. Hughes, unpublished observation), but one that is higher than previously reported for whole somites (Seger et al., 2011). Published values for Pax7^+^ cells per somite vary widely even at 24 hpf (Feng et al., 2006, Hammond et al., 2007, Hollway et al., 2007, Seger et al., 2011); our light fixation regime and long antibody incubations may explain the difference. The regional variations in MPC proliferation (reduced at horizontal myospetum), migration (primarily from vertical myosepta into myotome) and differentiation (highest in dorsal edge and deep myotome) that we describe show that MPC dynamics are balanced and locally controlled. Little is known of local signals in zebrafish larvae, although Fgf, Hh and Wnt signalling have been implicated in controlling DM behaviour at earlier stages (Feng et al., 2006, Hammond et al., 2007, Lewis et al., 1999, Tee et al., 2009). To understand muscle growth, it will be essential to elucidate how local signals affect myogenesis in discreet somitic regions.

### 4.3 Role of GSK3 signalling in myogenesis

Our small molecule screen revealed that inhibition of GSK3 using the ATP-competitor BIO prevents the normal and induced migration of a subset of MPCs into the myotome. GSK3 regulates several signalling pathways, including Wnt/ßcat. In vitro, BIO has been shown to maintain human embryonic stem cells in the pluripotent state and prevent their epithelial-mesenchymal transition by mimicking the action of the Wnt/ßcat pathway (Sato et al., 2004, ten Berge et al., 2011, Ullmann et al., 2008). Wnt signalling through GSK3ß can regulate DM behaviour in amniotes and Wnt signals also control muscle fibre patterning in the amniote somite (Gros et al., 2009, Hutcheson et al., 2009, Linker et al., 2005). Once myoblasts have formed, β-catenin-dependent Wnt signalling has been suggested to promote their differentiation (Brack et al., 2008, Jones et al., 2015), whereas non-canonical Wnt signalling may expand MPCs and promote their motility (Bentzinger et al., 2014, Le Grand et al., 2009). We observed a small but significant increase in fibre number in response to BIO, suggesting that some MPCs have differentiated to form new fibres. Thus, as in amniotes, GSK3ß activity may play different roles in successive stages of myogenesis (Lien and Fuchs, 2014).

In zebrafish, gain of function Wnt/β-catenin signalling caused by *axin1*/*apc1* double mutation has been shown to increase Pax3/7 cell proliferation, but without increase in Pax3/7 cell number (Tee et al., 2009). Congruently, we observe that BIO prevents entry of Pax7a^+^ cells into the myotome, also without a significant increase in dermomyotome cell number. These findings may be explained by the increased dermomyotomal apoptosis observed by Tee et al. (2009). Tee et al. also report increased apparent thickness of fibres in *axin1*/*apc1* mutants and LiCl-treated embryos. We observe no such effect with BIO applied at a somewhat later stage, or with LiCl treatment (Groves and Hughes, unpublished data). On the contrary, if anything, we observe a slight reduction in myotome volume by treatment with BIO alone, but no effect on the recovery of the myotome in *myod* mutants. Currently, we favour the simple interpretation that nuclear increase in *myod*^*fh261*^ mutant fast fibres arises from increased fusion of MPCs. A definitive test of this hypothesis will require ablation of the various MPC populations.

β-catenin was shown to be required for muscle hypertrophy fibre-autonomously following muscle overload in rodents (Armstrong and Esser, 2005, Armstrong et al., 2006). Our data show that BIO, which is expected to activate β-catenin signalling throughout the fish, does not alter developmental fibre growth. Thus, β-catenin signalling alone may be insufficient to promote fibre hypertrophy.

### 4.4 Robustness in myogenesis

The finding that Myod function limits muscle fibre size in larval zebrafish parallels data in mouse showing that Myogenin and Mrf4 can prevent atrophy or induce hypertrophy, respectively (Moresi et al., 2010, Moretti et al., 2016). This emerging theme suggests that a function of the duplicated vertebrate MRF genes within fibres is robust regulation of muscle size. Although Myod is well known to promote MPC terminal differentiation (Kablar et al., 1997, Yablonka-Reuveni et al., 1999), our data are inconsistent with a simple delay in myogenesis in *myod* mutants. Fibre number is reduced, but does not recover when excess MPCs subsequently differentiate, suggesting that a critical window for fibre formation has been missed. Moreover, ongoing new fibre formation is inhibited, raising the possibility that MPCs in specialised regions or with specific characteristics may be particularly vulnerable to lack of Myod function. Instead of a persistent defect, however, overall muscle size regulates to the normal value. This homeostasis is achieved by changing behaviour of both MPCs, through altered migration, and fibres themselves, by increasing volume per nucleus. At present, we cannot reject the hypothesis that two unrelated roles of Myod, firstly to drive myoblast differentiation to form new fibres and secondly to limit increase in fibre size, coincidentally cancel out to yield precisely the ‘correct’ myotome size. Nevertheless, the recent discovery of MPC diversity in the early zebrafish somite (Gurevich et al., 2016, Pipalia et al., 2016) raises the possibility that distinct MPC populations may preferentially contribute to hypertrophy in *myod* mutants. Such robustness may explain how stochastic variation in the low MPC numbers in each somite does not lead to maladaptive variation in muscle mass along the body axis or between left and right sides.

Our discovery of feedback regulation on muscle fibre size that can compensate for reduction in fibre number is intriguing in the light of control of tissue size in general. It suggests that skeletal muscles show ‘quorum sensing’, whereby tissue size is maintained at an appropriate set point. In myotomal muscle, such a process is essential to ensure the smooth change in body shape required for efficient hydrodynamics. Irrespective of fish size, age, nutrition or genetic background, each somite along the body axis has a precisely regulated size relative to its anteroposterior neighbours that yields the streamlined body shape. The difference in size of successive myotomes is small, yet perfectly graded, indicating great precision in the setting of myotome size. Such precision likely requires multiple semi-redundant feedback controls, perhaps explaining the lack, to date, of described mutants with an erratic myotome size phenotype. If similar quorum sensing controls other organ systems, our finding may have general importance in explaining animal growth and form.

In summary, we examined the role and regulation of Pax7^+^ MPCs in larval muscle growth. We observed tight regional control of MPC numbers, distribution and behaviour within the somite and myotome. Perturbations that alter muscle size and MPC number were rapidly corrected, suggesting the existence of a homeostatic mechanism that senses muscle size and ensures robust development in the face of environmental and genetic insults.

## Competing interests

The authors declare no competing interests.

## Author contributions

Experiments were performed by SDR (Figs. 1D;2;3;4C,D;5;7A,C-H;8A,B;S1-S4;S6;S7), VCW (Figs. 6;9;S5), TGP (Figs. 6F-H;7B;8A,C;9;S7), CLH (Fig. 1A-C), KL (Fig. 1E), SK (Fig. 4A,B) and RDK (Fig. 8A,B). RDK proposed and initiated the BIO experiments. SMH conceived the project, provided advice and wrote the manuscript with input from all authors.

## Funding

SMH is a Medical Research Council (MRC) Scientist with MRC Programme Grant G1001029 and MR/N021231/1 and Biotechnology and Biological Sciences Research Council (BBSRC) BB/K010115/1 support. CLH had a MRC PhD studentship. RDK was funded by BBSRC (BB/I025883/1) and Wellcome Trust (101529/Z/13/Z).
